# Seasonal drivers of faecal glucocorticoid metabolite concentrations in an African strepsirrhine primate, the thick-tailed greater galago (*Otolemur crassicaudatus*)

**DOI:** 10.1093/conphys/coab081

**Published:** 2021-10-25

**Authors:** Channen Long, Adrian Tordiffe, Michelle Sauther, Frank Cuozzo, James Millette, Andre Ganswindt, Juan Scheun

**Affiliations:** 1 Department of Paraclinical Sciences, Faculty of Veterinary Science, University of Pretoria, Onderstepoort, 0110, South Africa; 2 National Zoological Garden, South African National Biodiversity Institute, Pretoria, 0001, South Africa; 3 Department of Anthropology, University of Colorado, Boulder, CO 80309, USA; 4 Lajuma Research Centre, Louis Trichardt (Makhado), 0920, South Africa; 5 Department of Zoology and Entomology, Mammal Research Institute, University of Pretoria, Pretoria, 0028, South Africa; 6 Department of Life and Consumer Sciences, University of South Africa, Johannesburg, 1710, South Africa

## Abstract

As global non-human primate populations show dramatic declines due to climate change, land transformation and other anthropogenic stressors, it has become imperative to study physiological responses to environmental change in order to understand primate adaptability and enhance species conservation strategies. We examined the effects of seasonality on faecal glucocorticoid metabolite (fGCM) concentrations of free-ranging male and female thick-tailed greater galagos (*Otolemur crassicaudatus*) in an Afromontane habitat. To do so, we established an enzyme immunoassay (EIA) for monitoring fGCM concentrations in the species using a biological validation. Following this, faecal samples were collected each month over the course of a year from free-ranging males and females situated in the Soutpansberg Mountains, Limpopo, South Africa. Multivariate analyses revealed lactation period was a driver of fGCM levels, whereas sex and food availability mostly influenced seasonal fGCM concentrations in the total population. Thus far, the results of this study show that drivers of fGCM levels, an indication of increased adrenocortical activity, in *O. crassicaudatus* are numerous and complex within the natural environment. The species may be adapted to such conditions and an extreme change to any one component may result in elevated fGCM levels. This increases our understanding of strepsirrhine primate physiology and offers initial insights into species adaptability to a challenging environment.

## Teaser

Seasonal faecal glucocorticoid hormone concentrations in a free-ranging population of thick-tailed greater galagos (*Otolemur crassicaudatus*) suggests that changes associated with sex, reproductive state, food availability and ambient temperatures are reflected in faecal glucocorticoid metabolite patterns.

## Introduction

More than 60% of all non-human primate populations have been identified as highly susceptible to changes in the environment (Schloss *et al*., 2012; Schwitzer *et al*., 2019) and are consequently threatened with extinction (Estrada *et al*., 2017). Seasonal change can play an important role in the life history of different organisms, across varied habitats and time, and at least some primates will also experience naturally occurring environmental change at varying life history stages (Carré and Cheddadi, 2017). Environmental change influences reproductive success, availability of food resources and available habitat types (van Schaik and Brockman, 2005; Frederiksen *et al*., 2014; Campos *et al*., 2017). Similarly, reproductive state will affect an individual’s physiology (Rangel-Negrín *et al.,* 2009; Charpentier *et al*., 2018). A number of fields (such as behavioural and physiological) have been developed to monitor the effect of life history stages and environmental change in a given species (Homyack, 2010; Takeshita *et al*., 2018).

Behavioural (Pruetz and Bertolani, 2009) and hormone-based studies have been used to assess adaptability of animals to extrinsic stressors (environmental change/reproduction) to ensure their survival (Dettmer *et al*., 2012; Goymann, 2012). Naturally occurring seasonal change, such as temperature and rainfall variation, as well as fluctuations in resource availability (Feng *et al*., 2013), can induce physiological changes via the hypothalamic–pituitary–adrenal (HPA) axis leading to an increase in the production and secretion of glucocorticoids (GCs; Romero, 2002; Sapolsky, 2002). Environmental temperatures may have an inverse relationship with GC concentrations; for example, to maintain optimal body temperature, endotherms must increase their energy expenditure through the hyperactivation of the HPA axis (McKechnie and Wolf, 2019). Furthermore, behavioural attributes (such as decreased activity) will also be implemented to withstand the temperatures (Wingfield, 2013). In seasonal environments, there is a strong association between the dry season and increased GC concentration owing to food and water shortages (Davies *et al.,* 2013; Hernandez *et al*., 2018). For example, an increase in GC levels during seasonal dry periods ensures a constant source of energy through the metabolism of stored fat as shown in Magellanic penguins (*Spheniscus magellanicus*; Walker *et al.* 2005). However, once energy reserves are depleted, a decrease in GC production is found to maintain homeostasis. In addition to meeting energy requirements, elevated GC levels have also been found during periods of reproductive activity (Romero, 2002). For example, in male primates, the mating season seems to significantly influence GC concentrations as increased aggression and activity prevail as documented in red-fronted lemurs (*Eulemur fulvus rufus*) by Ostner *et al*. (2008). Whereas, in female primates, elevated GC levels are associated with the ovarian cycle: GC secretions are necessary both during the late gestation, owing to placental and foetal lung development (Bolt *et al*., 2001; Grier and Halliday, 2004), and lactation stages as these are considered energy costly (Cavigelli, 1999; Williams *et al*., 2007; Rimbach *et al*., 2013). There can also be sex- and age-related differences in the physiological stress response in a species. For example, wild ring-tailed lemurs (*Lemur catta*), responded differently to the ecological consequences of cyclones and droughts, with adult female’s response being more variable during droughts, adult male’s responses more variable during cyclones and subadults having higher cortisol values during cyclones (Fardi *et al*., 2017). An acute increase in GC concentrations can be adaptive in nature, leading to an increase in available energy and alterations in behaviour (Romero, 2002; Sapolsky, 2002). However, a chronic increase in GC concentrations can lead to the suppression of the immune response and reproductive abilities (McEwen, 1998; Crossin *et al.*, 2016; Fardi *et al*., 2017). Hence, assessing GC concentrations of free-ranging populations, as a model for stress-associated environmental alterations, has become an important technique to better understand the effect of stressors on individuals and populations and the likelihood of survival during challenging periods (Romero, 2002; Sapolsky, 2002; Beehner and McCann, 2008).

Non-invasive hormone monitoring, through the utilization of faeces, has become a preferred technique when studying wildlife. The collection of faecal samples requires little to no direct human–animal interaction, while allowing for longitudinal and repeated sampling (Heistermann, 2010; Touma and Palme, 2005). Furthermore, related hormone metabolite concentrations within a matrix like faeces are less affected by the episodic fluctuations of hormone secretions observed within blood (Vining *et al*., 1983; Russell *et al*., 2012). However, prior to the use of matrices like faeces for monitoring stress-related GC metabolite concentrations in a species for the first time, it is important to reliably investigate the applied test system to ensure suitable quantification of GCs for the species in question (Touma and Palme, 2005; Heistermann *et al*., 2006). It is widely accepted that the stimulation of the adrenal cortex via administration of adrenocorticotropic hormone (ACTH) adequately mimics a physiological stress response within an individual (Palme, 2005). However, conducting an ACTH challenge is often not possible due to the lack of accessibility to the species, the size of the individual, the research locality or their International Union for Conservation of Nature (IUCN) status. Therefore, the use of a biological validation has become increasingly popular as an alternative approach (Touma and Palme, 2005; Sheriff *et al*., 2011) in which individuals are exposed to a biological stressor (such as captivity or predation cues) known to activate the HPA axis, resulting in a rise in GC secretion (Kersey and Dehnhard, 2014). The use of non-invasive measures to monitor fGCM levels in response to external stressors has been successfully demonstrated in several strepsirrhine primate species including red-bellied lemurs (*Eulemur rubriventer*; Tecot, 2008) and ring-tailed lemurs (*L. catta*; Gabriel *et al.,* 2018). Studies focusing on the stress response associated with reproductive state and environmental challenges has been conducted in numerous primate species including yellow baboons (*Papio cynocephalus*; Gesquiere *et al*., 2008), pileated gibbons (*Hylobates pileatus*; Pirovino *et al*., 2011) and white-faced capuchins (*Cebus capucinus*; Carnegie *et al*., 2011). On mainland Africa, endocrine monitoring to determine adrenocortical activity has only been applied to one species of strepsirrhine primate, the African southern lesser bushbaby (*Galago moholi*; Scheun *et al*., 2015, 2016). Therefore, there is a need to increase our understanding of the influence of seasonality on the stress response in strepsirrhine primates.

The greater thick-tailed galago, *Otolemur crassicaudatus* (currently rated as Least Concern; IUCN Redlist; Masters and Génin, 2016), is an arboreal, nocturnal primate found in montane, riverine and evergreen forests of Botswana, Swaziland (Eswatini), Uganda, Kenya, Tanzania, Zambia, Angola, Zimbabwe, Mozambique and northern/north-Eastern South Africa (Masters and Génin, 2016). Within its distribution range, this species feeds predominantly on a variety of fruits, insects and tree exudates as available (Hladik, 1979; Clark, 1985; Happold and Happold, 1992; Nekaris and Bearder, 2011; Masters and Génin, 2016). *Otolemur crassicaudatus* is known to be polygamous (Bearder, 1974), with mating occurring during the Austral winter months (Eaton *et al*., 1973; Bearder, 1974). Female gestation length is estimated to be between 130 to 135 days (Manley, 1966; Bearder, 1974), resulting in the birth of one or two individuals during October and November (Nekaris and Bearder, 2011; Masters and Génin, 2016). Thus far, there is little information regarding their physiological responses to seasonality and reproductive activity. Therefore, in this study, a longitudinal hormonal monitoring in this species was conducted.

This study was conducted to monitor seasonal variation in GC hormone output in a free-ranging *O. crassicaudatus* population across differing seasonal environmental conditions. More specifically, this study (i) evaluated the suitability of four enzyme-immunoassays (EIAs) to measure faecal glucocorticoid metabolite (fGCM) concentrations in *O. crassicaudatus* by monitoring a handling event as a form of a biological validation and (ii) assessed correlated effects, such as reproductive period, climatic variables and food availability with fGCM concentrations over a longitudinal period.

This study hypothesized that (i) differences in fGCM concentrations between sexes will be notable; (ii) fGCM concentrations in males will be elevated during the mating season caused by increased competition-related aggression and activities; (iii) females will experience raised fGCM concentrations during the gestation and lactation periods owing to increased energy expenditure; and (iv) fGCM concentrations will be affected by the seasonal fluctuations in ambient temperatures, rainfall and food availability.

## Materials and methods

### Ethics statement

Ethical clearance to conduct the biological validation, including the capture and housing of animals, and seasonal analysis, requiring the capture and sampling of free-ranging animals, was received from the Research and Ethical Sciences Committee at the South African National Biodiversity Institute’s (SANBI) National Zoological Gardens (NZG; Project 18/26) and the Animal Ethics Committee of the University of Pretoria (Project V037-17).

### Study site

This study was conducted at the Lajuma Research Centre (23.0381° S, 29.4429° E), Soutpansberg mountains, Limpopo province, South Africa. Here, *O. crassicaudatus* individuals frequent the woodland and mist belt forest habitats within this highly seasonal, temperate, relatively high altitude (study area ranges from 1200 to 1400 m) environment (Hughes *et al*., 2005; Hahn, 2017). In addition to the diverse vegetation and food resources, the site also has few human settlements. The area sampled is ~3 km^2^, with individuals captured along one of the designated transects running in a general south-east to north-west direction (Phukuntsi *et al*., 2020). Importantly, *O. crassicaudatus* at this site appear to have large home ranges and certain individuals travelled for up to 2 km within the sampling range (personal observations: ML Sauther, FP Cuozzo). Some individuals observed within the study site appear to travel into the site but do not necessarily reside on the property (52 of the 94 distinct *Otolemur* individuals, 55%, captured 2013–2018 were only captured once; unpublished data: ML Sauther, FP Cuozzo).

### Weather data

For this study the seasons are referred to as winter (June–August), spring (September–November), summer (December–February) and autumn (March–May), with apparent dry and wet periods similarly defined by Nowack *et al*. (2013). Mean daily ambient temperature (°C), relative humidity (%), dew point (°C) and heat stress index (°C) were recorded from August 2017 to June 2018 using the Kestrel Drop D2 climate monitor and data loggers positioned throughout the study area (Kestrel© Instruments, Nielsen-Kellerman Co, PA, USA). Additional climate data were logged using a Davis Instruments sensor suite linked via wireless to a Davis Pro 2™ console (Davis© Instruments, CA, USA) and results were provided by the Ndlovu Node of the North-Eastern Mountain Observatories project of the South African Environmental Observation Network. The mean daily high and low temperatures (°C) and wind chill factor (°C) were calculated for each season, while a cumulative rainfall (mm) total for each day and month were calculated. All weather monitors were placed within the study site, near trapping locations to ensure on-site weather was recorded.

### Determining food availability

To assess insect availability two light traps were set up near the field campsite (trap 1: 23°02′19.6″S 29°26′34.8″E; trap 2: 23°02′20.4″S 29°26′34.6″E), ~20 m from any other source of light, from September 2017 to June 2018. A nylon lamp was secured <1 m from the ground, with a catching container placed below the light. The second light and catching container were secured on a tree branch ~3 m above the ground. Both lights remained in the same location for the entirety of the monthly sampling periods. The lights were turned on prior to sunset (17:00–19:15) and turned off prior to sunrise (04:30–06:00) each day. Once captured, the number of invertebrates and order and/or common name classification, if possible, was recorded. Insects were then stored in vials containing 70% ethanol or 90% isopropanol. In addition to these insect captures, *O. crassicaudatus* faeces (*n* = 65), collected during the monthly animal captures from August 2017 to June 2018 (see below), were assessed for insect and seed abundance. Individually identified freeze-dried faecal samples were dissected and seed and insect parts separated. All seeds were counted with the presumption that each seed represented one fruit, except for fig (*Ficus* sp.) fruit, which constitute numerous, tiny seeds. The insect remains were measured in a 1.7-ml vial to the nearest 0.1 ml. The remains were identified if possible, and the volumes of the content were recorded for each sample. Additionally, monthly collections of gum from all trapping sites were conducted to assess gum availability from July 2017 to June 2018. Gum samples were removed from trees using a sharp knife, avoiding removal of tree bark and once transferred to the laboratory, were weighed in grammes. Trees were selected based on visible presence of gum; however, as observations of *O. crassicaudatus* feeding activities increased, more trees were added (personal observations: C Long, JB Millette). The gum samples were stored in a frozen state for future nutritional content analysis.

### Animal captures

Captures were conducted once a month from May 2017 to June 2018 utilizing a total of 48 traps, which were set-up throughout the study site. In addition to the monthly captures, a more comprehensive capturing period was conducted over 7 days approximately every 3 months. These sampling seasons were used to identify different reproductive periods based on Bearder (1974) and Masters *et al*. (1988) and Millette *et al*. (personal observations): June 2017 (mating 1), September 2017, January 2018 (lactating), March/April 2018 (post-lactating) and May/June 2018 (mating 2). These interval events were accompanied by a certified veterinarian to perform health checks and confirm reproductive status on the captured individuals.

Throughout the study an attempt was made to trap and sample a minimum of 20 adult animals (10 males, 10 females) every month. Havahart® traps (Woodstream Corporation, 43 × 17.8 × 17.8 cm, Lititz, PA, USA) were placed into designated trees and baited with a mixture of peanut butter and bananas. Traps were set at sunset (17:00–18:30) and checked at sunrise (05:00–07:00). Previous sampling periods determined the individuals were more visibly stressed (for instance, pacing) when traps were checked throughout the night prior to being transported to the field lab area for sampling; thus individuals were kept in the traps throughout the night surrounded by familiar sounds and conditions. Furthermore, previous camera trap evidence indicated multiple periods of *O. crassicaudatus* activity throughout the night (personal communication, ML Sauther, FP Cuozzo). Only fresh samples were collected, namely, evident of moist, soft texture, further eliminating the possibility that old samples were collected. Samples that fell from the trap or were contaminated with urine were not analysed in this study. Traps were cleaned with a mixture of isopropanol (90%) and water after each successful capture. Additionally, all fresh faeces collected from an individual in one night (constituting one sample) were pooled together during the extraction procedure to avoid any effects of GC diurnal cyclicity.

When captured, an individual was removed from the trap using a carrier bag, where it was weighed, sexed, scanned for a passive identification transponder (see below), to determine the animal’s identity, and then released at the site of capture. All faecal samples within the trap were collected and stored at −20°C until analysis.

During the comprehensive captures, the project veterinarian assessed the health, and confirmed the age-class and reproductive status of all captured individuals. Pregnancy was determined by abdominal palpation in September 2017, and lactation confirmed in January 2018. However, some females were not recaptured (7 out of 20 different individuals), and therefore their reproductive status could not be confirmed. The status of the vulva (open/closed; if open this indicates receptive to mating) was noted, and the length of the vulva and nipple in females were measured to determine reproductive maturity and lactation (e.g. long nipples are associated with adult lactating females). In males, testes length and width were measured to support confirmation of reproductive status (e.g. large testes are associated with the mating season). Males captured in other sampling periods (when females are categorized as lactating/post-lactating) of the year were identified under the ‘non-mating’ period. All individuals captured for the first time were injected subcutaneously with a passive identification transponder (ID100 Trovan®, EURO I.D., Weilerswist, Germany) to determine individual identity during future captures.

### Biological validation experiment

A biological validation (handling event) was conducted in May 2018 using one known male and female from the free-ranging population to establish a method for measuring fGCM concentrations, as an indication of adrenocortical activity, in *O. crassicaudatus.* As no facility within South Africa could provide captive *O. crassicaudatus* for the validation process at the time of the study, a male and female from the free-ranging population at the Lajuma Research Station were used.

The veterinarian confirmed the female was not pregnant or lactating at the time of capture. Once all morphometric samples were collected, both individuals were housed in separate outside cages (350 × 120 × 230 cm) for a total of 7 days. To remove the possibility of environmental contamination of faecal samples, a sheet of tarpaulin was placed at the bottom of each cage to catch the faecal samples and allow the run-off of urine. A black tarpaulin sheet and shade cloth were used to cover the top of the cage to limit sunlight during the day. Branches were placed throughout each cage and a makeshift nest was hung in a dark corner for the comfort of both individuals. The cages were placed in an area away from human activity to decrease anthropogenic stressors on the animals. Individuals were fed a diet of available fruit, dry cat food pellets (Catmor®, South Africa) and peanut butter. Water was available *ad libitum*. A veterinarian ensured the health of the study animals throughout the validation period.

Both individuals were left to acclimatize to the new environment for 2 days. During this period, no samples were collected and human interaction was limited to reduce possible psychological distress. The nature of the validation process and the need for a registered veterinarian on-site during the process limited the time available for animal acclimation. On Days 3 and 4, faecal samples were collected hourly during the night (18:00–05:00) and bi-hourly during the day (06:00–18:00) to determine baseline fGCM concentrations of both individuals. At 18:00 on Day 4, individuals were captured in baited Havahart® traps within the enclosure, and then were appropriately held by the back of the neck and pelvic region for 2 minutes to prevent any risk of injury to the animals by the veterinarian in order to facilitate a stress response. The entire process was completed in less than 5 minutes. Sample collection continued for a further 72 hours until 18:00 on the seventh day when both individuals were released at their respective site of initial capture. Samples were collected by reaching into the enclosures through a mesh opening, while preventing additional distress on the individuals. Faecal samples were collected by using sterilized forceps and placed into a 1.5-ml microcentrifuge tube, labelled and stored at −4°C within 20 minutes of collection. Samples were kept frozen until reaching the Endocrinology Laboratory of the SANBI National Zoological Garden Pretoria.

Although fGCM patterns may have been elevated during the acclimation period, the aim of this validation experiment was to determine whether one of the available EIAs could measure an increase in fGCM levels (and adrenocortical activity) following a stressful event. The results of the validation process confirmed that the chosen EIA could do so.

### Faecal steroid extraction and analysis

Sample extraction was conducted following the methods used by Fieß *et al*. (1999). Faecal samples were lyophilized, pulverized and sieved through a thin mesh to remove non-faecal matter. Subsequently, 1.5 ml of 80% ethanol was added to 0.050–0.055 g of faecal powder and vortexed for 15 minutes, before being centrifuged at 1500 x *g* for 10 minutes. The supernatant was then transferred to a clean 2.5-ml microcentrifuge tube and stored at −20°C until analysis.

A total of 30 faecal samples from the validation experiment were extracted for analysis: (pre-handling) male = 5 samples, female = 5 samples; (post-handling) male = 10 samples, female = 10 samples. Faecal extracts resulting from the biological validation process were measured for immunoreactive fGCM concentrations using four EIAs: (i) oxoaetiocholanolone I (detecting 11,17 dioxoandrostanes), (ii) oxoaetiocholanolone II (detecting fGCMs with a 5β-3α-ol-11-one structure), (iii) cortisol and (iv) 11β-hydroxyaetiocholanolone. Details of the assays, including cross-reactivities, are described by Palme and Möstl (1997) for oxoaetiocholanolone I, Möstl *et al.* (2002) for oxoaetiocholanolone II and cortisol and Frigerio *et al*. (2004) for 11β-hydroxyaetiocholanolone. The test of parallelism confirmed serial dilutions of faecal extracts gave displacement curves that were parallel to the respective standard curve (slope, <4%). Inter-assay coefficients of variation (CV) of high- and low-value quality controls were (i) 11.72% and 11.75% for oxoaetiocholanolone I, (ii) 10.70% and 10.97% for oxoaetiocholanolone II, (iii) 12.20% and 14.38% for cortisol (combined both validation and seasonal CVs) and (iv) 6.13% and 14.47% for 11β-hydroxyaetiocholanolone. Two plates were used for each EIA for the inter-assay CVs. The intra-assay CV of high- and low-value quality controls were (i) 5.65% and 6.11% for oxoaetiocholanolone I, (ii) 2.15% and 2.21% for oxoaetiocholanolone II, (iii) 5.67% and 6.90% for cortisol and (iv) 7.43% and 6.96% for 11β-hydroxyaetiocholanolone. The sensitivities of the EIAs used were 0.6 ng/g DW for all assays except 11β hydroxyaetiocholanolone, which was 1.2 ng/g DW. Assays were conducted on microtiter plates as described by Scheun *et al*. (2016).

For the seasonal fGCM evaluation, 185 samples from 24 male (total samples = 76, 3.44 ± 2.58 SD samples/individual) and 21 female (total samples = 109, 5.30 ± 5.36 SD samples/individual) free-ranging individuals were analysed using the cortisol EIA (see Results: Biological validation).

### Data analysis

#### EIA validation

To calculate baseline fGCM concentrations during the biological validation experiment, an iterative process was implemented that excluded baseline samples greater than the mean plus 1.5 SD for both *O. crassicaudatus* individuals (Ganswindt *et al*., 2014; Scheun *et al*., 2015). Here, all fGCM concentrations collected before and after the handling stressor greater than the mean plus 1.5 SD were excluded, the average recalculated and the process repeated until no values exceed the new mean (the baseline) plus 1.5 SD. To determine the effect of handling, the absolute change in fGCM concentrations was determined by calculating the quotient of baseline and post-handling peak fGCM samples. Subsequently, a 100% (1-fold) increase in the calculated response indicated baseline value and not a change in HPA response (as demonstrated in Scheun *et al*., 2018).

**Table 1:** The mean baseline ***±*** SD (ug/g DW), baseline +1.5 SD peak values (ug/g DW) and the change from the baseline peak percentage (%) determined for the male (*n* = 1) and the female (*n* = 1) *O. crassicaudatus* individuals during the biological validation

Male				
	Oxoaetiocholanolone I	Oxoaetiocholanolone II	Cortisol	11β-hydroxyaetiocholanolone
Baseline + 1.5 SD	0.07	0.27	2.15	0.79
Peak (ug/g DW)	0.11	0.67	14.72	3.28
Peak response (%)	57.14	149.22	**584.82**	312.83
Female				
Baseline + 1.5 SD	0.10	0.30	4.43	1.69
Peak (ug/g DW)	0.10	0.65	11.89	2.50
Peak response (%)	71.83	117.95	**168.41**	48.06

Bolded percentages indicate the highest percentage peak change from baseline.

#### Seasonal analysis

All statistical analyses were conducted in R (R Core Team, 2020). All fGCM concentrations were presented as nanograms of immunoreactive hormone metabolites per gramme of dry faecal powder weight (ng/g DW). *P*-values of <0.05 were deemed significant.

Seasonal fluctuations (winter, spring, summer, autumn) in food availability were assessed by conducting analysis of variance (ANOVA) tests on insect counts from the light traps, faecal seed counts and faecal insect volumes. ANOVA tests were conducted on the seasonal values for rainfall and ambient temperature to determine any significant fluctuations. Mean fGCM concentrations (± standard deviation) were determined separately for males and females.

Covariates of free-ranging male and female fGCMs were assessed by analysing the variation of fGCM concentrations from 25 male (*n* = 76) and 20 female (*n* = 109) individuals. Linear mixed models (LMMs) were used to explore the variation in fGCM hormone response within the population in response to annual seasonal effects using the *lmer* function from the ‘lme4’ package (Bates *et al*., 2014). Global model sets were used. After using Q-Q plots to visually assess that normality of the data met the assumptions of the model and, log-transformed fGCM concentrations were used as the response variable. All quantitative fixed effects were z-transformed (Schielzeth, 2010) for more accurate model fittings and to facilitate model estimate comparisons. Two models were used for analysis as reproductive status was only confirmed in individuals sampled during the comprehensive sampling including health checks. In the first model the effects of environmental and dietary factors were assessed: sex, ambient temperature (°C), rainfall (mm), insect count, seed count and gum availability were used as fixed effects. In the second model, the influence of each reproductive status (lactating, mating 1, mating 2, post-lactating) on fGCM concentrations was described. For both models, Individual ID was used as the random effect to avoid pseudo-replication (Millar and Anderson, 2004). Collinearity was checked by determining variance inflation factors (VIFs; variables were excluded if VIF > 3; Field, 2009; Neter *et al*., 1996; Dias *et al.,* 2017; Chaves *et al.,* 2019) using the *vif* function from the ‘car’ package applied to standard linear models for both sexes excluding the random effects (Fox and Weisberg, 2011). Models were selected using the *dredge* function in ‘MuMIn’ package (Barton, 2018). The candidate models were ranked using AIC small sample correction (ΔAICc; Anderson, 2007). Marginal (m*R*^2^) and conditional *R*-squared (c*R*^2^) values were calculated for each model, explaining variance by the random (m*R*^2^) and the random and fixed variables (c*R*^2^), to justify the model selection (Nakagawa and Schielzeth, 2013; Nakagawa *et al*., 2017). R^2^ values were determined using the *rsquared* function in the ‘piecewiseSEM’ package (Lefcheck*,* 2016). Following the method suggested by Grueber *et al.* (2011), model averaging was performed, considering models with ΔAIC_C_ < 2 to have a strong support. The *confint* function was used to determine confidence intervals. Likelihood ratio tests were conducted (using the *anova* function in the ‘car’ package) to assess whether each selected model performed better when compared to the null model.

## Results

### EIA validation

**Figure 1:**
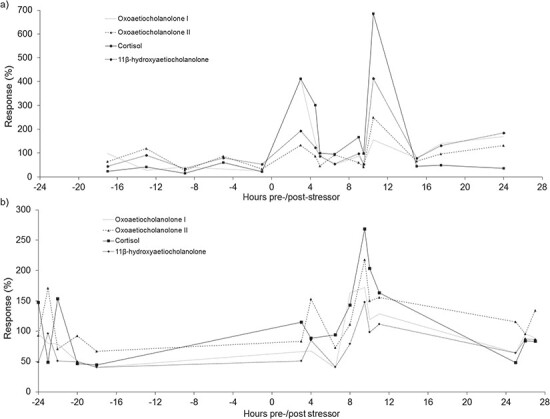
The (a) male and (b) female *O. crassicaudatus* fGCM response (%) for each EIA before and after the stressor (handling event). Time zero indicates handling event.

All four EIAs showed a considerable increase in fGCM concentrations following the handling event (Table 1). However, in the male it was the cortisol EIA that showed the highest peak fGCM increase (585%) at 10.5 h post-handling (Table 1; Fig. 1a.). Similarly, the cortisol EIA also showed the highest fGCM increase (168%) 9.5 h post-handling in the female (Table 1; Fig. 1b). As such, the cortisol EIA was chosen as the most suitable assay for monitoring fGCM concentrations in both sexes of *O. crassicaudatus*.

### Seasonal variation in food abundance

A total of 3820 insects were collected from the light traps from September 2017 to June 2018 (185 nights). Overall, there were significant seasonal differences in insect abundance (ANOVA: F_(287)_ = 6.89, *P* = 0.002), specifically between autumn and spring (Tukey honest significant [HSD]: *P* = 0.019; Table 2), and autumn and summer (Tukey HSD: *P* = 0.001).The lowest number of insects were captured during the winter period and was significantly different to summer months (Tukey HSD: *P* = 0.038).

**Table 2:** Measurements of food availability for *O. crassicaudatus* collected

Season	Insect count	Faecal seed count	Mean (±SD) faecal insect volume (ml)	Mean (±SD) gum density (g)
Winter	27 (14)	12 (3)	0.50 ± 0.21	0.45 ± 0.04
Spring	1212 (73)	281 (12)	1.23 ± 0.42	0.45 ± 0.04
Summer	1793 (96)	361 (13)	3.48 ± 1.98	1.34 ± 0.18
Autumn	747 (108)	1378 (37)	2.21 ± 1.43	0.78 ± 0.06

Insects were trapped in light traps, and their number counted, from September 2017 to June 2018. Subsequently, the number of seeds and insect content in faeces (ml) for each month were counted and weighed, respectively, from a subset of the total faecal samples. Gum density (g) was determined by weighing the samples collected from July 2017 to June 2018. Gum and faecal samples were not collected in December 2017. Sample size is denoted by the number in brackets following the total value. Mean values are followed by standard deviation values.

A total of 2032 seeds were recovered from 65 faecal samples. The majority of seeds found in faeces originated from fig (*Ficus* sp) and jojoba (*Simondsdia chinensis*) fruits. The highest number of seeds collected were during the autumn season (Table 2); however, the analysis of faecal seed count revealed no significant differences between seasons (ANOVA: *F*_*(*61)_ = 0.52; *P* = 0.67).

A total volume of 18.27 ml of insect content was recovered within the faecal samples. Documentation of insects in the faeces was limited as identification was difficult but social insects such as termites and ants were present. The analysis of faecal insect content was significantly lower in autumn (0.18 ± 0.29 SD) compared to the summer months (0.54 ± 0.60 SD; F_(61)_ = 3.22, *P* = 0.029; Tukey HSD: *P* = 0.016).

*Vachellia karroo* is the only tree species from which *O. crassicaudatus* individuals were observed consuming gum. Total gum volume of 182.7 g from 257 samples were retrieved and a significant difference in seasonal volume was determined (F_(253)_ = 12.48, *P* < 0.001). Gum density measured in summer was significantly higher than in autumn (Tukey HSD: *P* = 0.010), spring (Tukey HSD: *P* < 0.001) and winter (Tukey HSD: *P* < 0.001; Table 2).

### Seasonal weather variations

**Figure 2:**
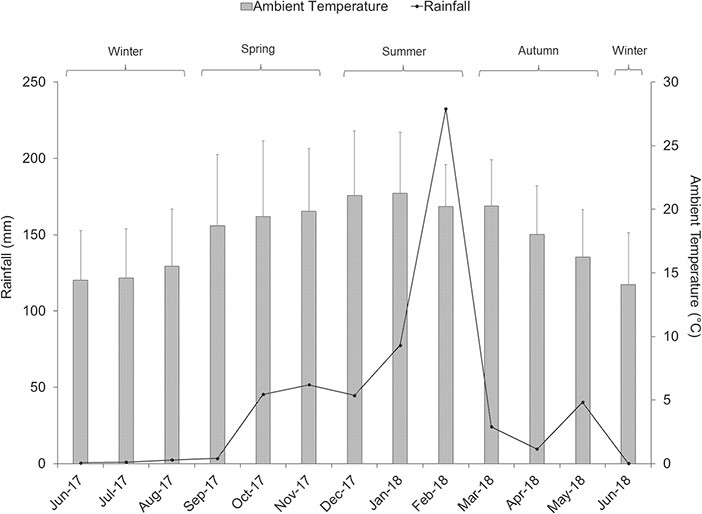
Cumulative monthly rainfall (mm) and mean monthly ambient temperature (°C) throughout the sample period (June 2017 to June 2018) for Lajuma Research Station in the Soutpansberg Mountains, Limpopo.

Seasonal differences were determined for rainfall and ambient temperature. Rainfall peaked in February 2018 (232 mm) with a significant rise in the summer months, compared to winter (F_(9)_ = 3.45, *P* = 0.065; Tukey HSD: *P* = 0.05) in which the study site received <1 mm of precipitation (Fig. 2). Ambient temperatures were significantly warmer in the spring (F(9) = 21.49, *P* = 0.001) and summer months (range = 11.3–34.0°C; Tukey HSD: *P* < 0.001) than in winter (range = 4.4–29.6°C; mean = 14.85 ± 0.59°C; Fig. 2).

### Seasonal reproductive differences

No significant differences were seen in adult male fGCM levels between the reproductive states (ANOVA: F_(27)_ = 1.92, *P* = 0.153) with only slight fGCM elevations during the non-breeding period (mean = 0.43 ± 0.29 ug/g DW; Fig. 4). Additionally, testes length also exhibited no significant difference across the reproductive periods; however, a slight increase in testicle length was measured in both mating periods (mean length: mating 1 = 2.46 ± 0.53 cm; mating 2 = 2.40 ± 0.19 cm) compared to the non-breeding period (mean length = 1.89 ± 0.51 cm). Adult female fGCM levels during the lactating period were significantly higher than during the mating 1 (ANOVA: F_(37)_ = 4.65, *P* = 0.008; Tukey HSD: *P* = 0.014) and post-lactating period (Tukey HSD: *P* = 0.043; Fig. 4); however, they did not significantly differ from the mating 2 period (Tukey HSD: *P* = 0.580). Furthermore, nipple length increased significantly in lactating period (ANOVA: F_(19)_ = 5.46. *P* = 0. 015) between mating 1 (Tukey HSD: *P* = 0.018) and mating 2 (Tukey HSD: *P* = 0.031); however, vulva length expressed no significant changes across the reproductive periods (ANOVA: F_(21)_ = 0.25, *P* = 0.785).

### Seasonal variation in fGCM concentrations

Overall, fGCM concentrations differed significantly between females (*n* = 109, mean = 0.55 ± 0.45 ug/g DW) and males (*n* = 76, mean = 0.34 ± 0.29 ug/g DW; *t*(181) = 3.55 *P* < 0.001). There was no seasonal difference in fGCM concentrations in male individuals (F(72) = 0.40, *P* = 0.751). Female fGCM concentrations were significantly different between seasons: F(104) = 2.83, *P* = 0.04; specifically, summer (*n* = 14, mean = 0.86 ± 0.56 ug/g DW) was significantly higher than winter (*n* = 27, mean = 0.46 ± 0.41 ug/g DW; Tukey HSD: *P* = 0.03) and autumn (*n* = 45, 0.51 ± 0.43 ug/g DW; Tukey HSD: *P* = 0.049).

**Figure 3:**
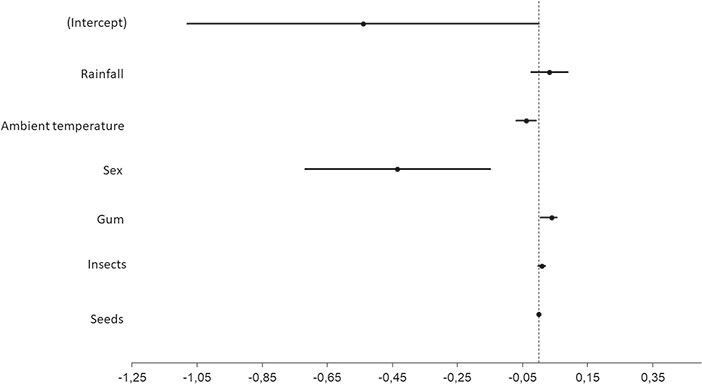
Coefficient plot depicting the beta coefficients (β) for each parameter in the model selected *O. crassicaudatus* fGCM concentrations. These parameters indicate a negative or positive influence on the fGCM concentrations. The error bars extending from the marker indicate 95% confidence intervals.

**Figure 4:**
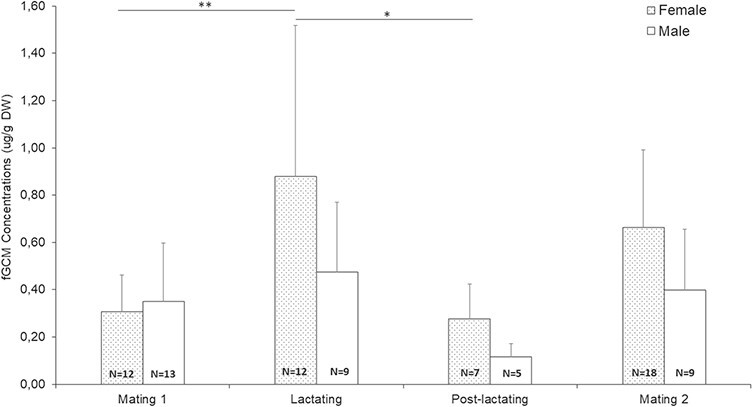
Mean fGCM concentrations for male and female *O. crassicaudatus* populations depicted by the allocated reproductive periods (mating 1, June–July 2017; lactating, January 2018; post-lactating, March 2018; mating 2, May–June 2018). Bars indicate the standard deviation for values for each season. Significant differences in fGCM concentrations between periods are illustrated by bar and asterisk (^*^P < 0.05, ^**^P < 0.01, ^***^P < 0.001). Sample size is indicated by *N*.

The models revealed significant effects of sex ($\square$^2^(1) = 9.56, *P* = 0.002, R_m/c_ = 0.08/0.19), insects ($\square$^2^(1) = 4.74, *P* = 0.03, R_m/c_ = 0.02/0.20), gum availability ($\square$^2^(1) = 9.27, *P* = 0.002, R_m/c_ = 0.04/0.23; Table 3; Fig. 3) and lactating reproductive status ($\square$^2^(3) = 23.62, *P* = 0.001, R_m/c_ = 0.22/040; Table 3; Fig. 4) on fGCM concentrations. The best-fit model included the variables sex, gum availability, ambient temperature and insect availability ($\square$^2^(7) = 26.58, AICc = 417.58, R_m/c_ = 0.16/0.26; Table 4); however, no significant effect of ambient temperature was determined ($\square$^2^(1) = 3.2044, *p* = 0.073, R_m/c_ = 0.02/0.18; Table 3).

**Table 3:** Model-averaged LMMs of faecal GC hormone variation in free-ranging *O. crassicaudatus*

Predictors	(β)	SE	ChiSq	*P*	95% CI
Environmental					
Intercept	−0.5397	0.2737			−1.081, 0.0001
Rainfall	0.0330	0.0282	0.8516	0.356	−0.023, 0.089
Ambient temperature	−0.0388	0.0155	3.2044	0.073	−0.069, −0.008
Sex	−0.4344	0.1421	**9.5632**	0.002	−0.718, −0.150
Gum	0.0300	0.0124	**9.2699**	0.002	0.006, 0.055
Insects	0.0087	0.0053	**4.7379**	0.029	−0.002, 0.019
Seeds	0.0005	0.0008	0.9847	0.321	−0.001, 0.002
Reproduction					
Intercept	0.425	0.049			0.325, 0.526
Lactating	0.253	0.074	**23.622**	0.001	0.105, 0.401
Mating1	−0.132	0.079		0.100	−0.290, 0.026
Mating 2	0.106	0.070		0.134	−0.034, 0.246
Sex	0.106	0.046	**2.825**	0.025	0.014, 0.197

Two global models were used to firstly identify the main environmental parameters (rainfall, ambient temperature, food availability and sex) and, second, the reproductive stages influencing fGCM concentrations. Parameters shown are model-averaged parameter estimates (β), standard error (SE), chi-squared values (ChiSq) and corresponding *P*-values and 95% confidence intervals (CI). Parameters exhibiting significant effects are shown in bold.

**Table 4:** Model selection results for averaged mixed effects models with modelling fGCM levels in free-ranging *O. crassicaudatus* individuals (*n* = 185)

Model: log~	*mR* ^2^	*cR* ^2^	df	AICc	ΔAICc	*w_i_*	ChiSq
Gum + Ta + Sex + Insects + (1|ID)	0.16	0.26	7	417.58	0.00	0.37	26.58^***^
Gum + Ta + Sex + (1|ID)	0.15	0.25	6	418.36	0.78	0.25	23.63^***^
Gum + Ta + Sex + Insects + Rainfall + (1|ID)	0.16	0.27	8	418.50	0.92	0.23	27.84^***^
Gum + Ta + Sex + Insects + Seeds + (1|ID)	0.16	0.26	8	419.52	1.94	0.14	26.82^***^

Individual ID was used as the random effect in all the models (1|ID). For each model, we report marginal (mR^2^) and conditional R-squared (cR^2^) values, number of parameters (K), Akaike’s Information Criterion with small sample correction (AICc), AICc distance from the best model (Δ), Akaike weight (w_i_) and likelihood ratio tests (ChiSq) comparing the null models to the selected models.

Ta, ambient temperature.

^***^ *P* < 0.001 indicates significant difference between selected model and the null model.

## Discussion

### EIA validation

This is the first study to successfully monitor seasonal fGCM concentrations as an indication of adrenocortical activity, in *O. crassicaudatus* using a validated EIA. Our study, therefore, attests to the use of this method for validating EIAs in wild individuals and provides new insight into the seasonal physiological responses of this nocturnal African primate species. The use of a biological stressor (capture, captivity and physical restraint) sufficiently induced an acute stress response in the *O. crassicaudatus* individuals, resulting in elevated fGCM levels. Similar approaches for establishing EIAs for measuring fGCM concentrations have been successfully used in several mammal species, for instance the grey mouse lemur (*Microcebus murinus*; Hämäläinen *et al*., 2015) and southern elephant seals (*Mirounga leonina;*  Engelhard *et al*., 2002). The results indicate a second peak prior to the highest peak in the cortisol EIA in both the male and female data 3 hours after the handling stress occurred. This could be due to an extrinsic event or stressor such as another animal nearing the enclosures. As the enclosures are situated outside, the exact cause of fGCM elevation cannot be accounted for.

### Sex differences in individual baseline fGCM values

During the validation, we report a considerable difference between the male and female individual baseline fGCM values, with the female expressing higher fGCM levels. Such differences have been reported in other primate species such as the pied tamarin (*Saguinus bicolor*; Armstrong and Santymire, 2013) and the common marmoset (*Callithrix jacchus*; Johnson *et al*., 1996). This may be a result of sex-related differences in metabolic processes and dietary differences altering gut passage time (Goymann, 2012) or that female adrenocortical activity may be elevated in this species. Differences in metabolic processes and reproductive activities could be factors contributing to these changes (Millspaugh and Washburn, 2004; Goymann, 2012).

### Reproduction

More energy-demanding reproductive conditions, such as lactation, could also cause significant changes to the concentration of hormone metabolites secreted (Boonstra *et al*., 2001). Sex differences in fGCM concentrations can be caused by a number of factors associated with reproduction (such as, gonadal steroid hormone synthesis; Handa *et al*., 1994; Cavigelli *et al*., 2003). In the results, females exhibited greater fGCM concentrations than males in all reproductive states except mating 1. Male primates can increase their GC secretions during the mating period where they exhibit greater aggression and may expend more energy such as seen in ring-tailed lemurs (*L. catta*; Starling *et al*., 2010), Verreaux’s sifakas (*Propithecus verreauxi*; Fichtel *et al*., 2007) and collared brown lemurs (*Eulemur collaris*; Balestri *et al.*, 2014). In contrast, primates who exhibit a low degree of mate competition, such as the tufted capuchin monkey (*Cebus apella*; Lynch *et al*., 2002), red-bellied lemurs (*E. rubriventer*; Tecot *et al*., 2013) and rhesus macaques (*Macaca mulatta*; Higham *et al.*, 2013), show limited no HPA hyperactivation.

The models revealed lactating reproductive status as a driver of adrenocortical activity in female *O. crassicaudatus*. In this study, females in the lactating period had the highest fGCM concentrations and the model revealed lactation state to have an influence on fGCMs. Energy-demanding reproductive conditions, such as lactation, could cause significant changes to the concentration of hormone metabolites secreted (Boonstra *et al*., 2001) for mammals (McLean and Smith, 1999) and specifically primates (Dufour and Sauther, 2002) and is often associated with elevated GC concentrations when compared to other non-reproductive stages (Rimbach *et al.*, 2013; Charpentier *et al.*, 2018). An increased secretion of GC may assist in mobilizing energy reserves during the lactation process (Romero and Wingfield, 2015) as reported in rhesus macaques (*Macaca mulatta*; Maestripieri *et al.,* 2008). Therefore, elevated female fGCM levels in the summer period could reflect lactation and increased energy expenditure owing to offspring protection (Ostner *et al*., 2008), as reported in chacma baboons (Beehner *et al*., 2005). It must be noted that elevated GC hormones may also be affected by other physiological activities. For instance, GC hormone secretion may increase in consequence of the development of foetal organs and adrenal gland during the late gestation phase (Ishimoto and Jaffe, 2011; Huang *et al.,* 2012). Furthermore, inter-female competition and aggression for resources has been shown to elevate GC secretions within animals (Creel *et al*., 2013), previously demonstrated in *G. moholi*, (Scheun *et al*., 2015), and could contribute to the elevated female fGCM concentrations during summer.

No significant increase was found in male fGCM levels and testes size during the mating period. Contrasting patterns have been observed in several group-living strepsirrhine species in which males will express significantly elevated fGCMs during the mating season, as seen in male ring-tailed lemurs (*L. catta*; Pride, 2005), Verreaux’s sifakas (*P. verreauxi*; Fichtel *et al*., 2007) and brown-collared lemurs (*Eulemur collaris*; Balestri *et al.*, 2014). This could indicate *O. crassicaudatus* males were not psychologically or energetically affected by the type of inter-male competition seen in strepsirrhine primates that live in complex social groups, such as *L. catta* (Sauther, 1991)., or the sample size in this study was too small or spermatogenesis is occurring in this species in the absence of testis size increases.

### Insects and gum availability

In this study gum availability was shown to influence fGCM values. Gum density decreased in the colder temperatures and increased during the summer months. Exudates are an essential food source for *O. crassicaudatus* (Bearder, 1974); as such, it is to be expected that a lower-quality diet may indicate individuals have fewer energetic resources and thus higher levels of fGCMs to help combat this metabolic challenge (Beehner and McCann, 2008). Insects are an ideal energy source comprised of fats, protein and micronutrients (Rumpold and Schlüter, 2013; O’Malley and McGrew, 2014). For *O. crassicaudatus*, insects most likely play an important role in meeting energy demands that gum and fruits cannot provide, especially in the warmer months during high insect density. A dramatic decline in insect populations was observed during the winter season (low ambient temperature and rainfall). However, from the results, low fGCM levels during the winter (low food availability) may indicate that this species is capable of finding an alternative method of coping, either by adjusting their metabolic rate or by opportunistically feeding on available food sources such as fruit. Nevertheless, insects can provide an important source of both protein and energy if they can be eaten in sufficient quantities (Rothman *et al.*, 2014).

## Conclusion

This study not only successfully validated the most appropriate EIA for monitoring fGCMs in *O. crassicaudatus* but also implemented the technique in a free-ranging population to facilitate determining the drivers of adrenocortical activity within the natural environment. This study shows that seasonal changes in sex, insect and gum availability, as well as reproductive state were found to influence GC output. From the results it is apparent that energetic consequences are a major factor contributing to GC secretion, namely reproductive state and food availability. This indicates that GC assessments constitute as a reliable tool to monitor the responses of this species to changes in their environment and opens the way to further research to enhance our understanding of species-specific responses to environmental and biological changes.
